# A machine learning-based PET/CT model for automatic diagnosis of early-stage lung cancer

**DOI:** 10.3389/fonc.2023.1192908

**Published:** 2023-09-15

**Authors:** Huoqiang Wang, Yi Li, Jiexi Han, Qin Lin, Long Zhao, Qiang Li, Juan Zhao, Haohao Li, Yiran Wang, Changlong Hu

**Affiliations:** ^1^ Department of Nuclear Medicine, Shanghai Pulmonary Hospital, Tongji University School of Medicine, Shanghai, China; ^2^ Shanghai miRAN Biotech Co. Ltd, Shanghai, China; ^3^ Department of Geriatrics, Ruijin Hospital, School of Medicine, Shanghai Jiaotong University, Shanghai, China; ^4^ Faculty of Business and Economics, University of Hong Kong, Hong Kong, China; ^5^ School of Life Sciences, Fudan University, Shanghai, China

**Keywords:** PET/CT, pulmonary nodule, lung cancer, diagnosis, machine-learning

## Abstract

**Objective:**

The aim of this study was to develop a machine learning-based automatic analysis method for the diagnosis of early-stage lung cancer based on positron emission tomography/computed tomography (PET/CT) data.

**Methods:**

A retrospective cohort study was conducted using PET/CT data from 187 cases of non-small cell lung cancer (NSCLC) and 190 benign pulmonary nodules. Twelve PET and CT features were used to train a diagnosis model. The performance of the machine learning-based PET/CT model was tested and validated in two separate cohorts comprising 462 and 229 cases, respectively.

**Results:**

The standardized uptake value (SUV) was identified as an important biochemical factor for the early stage of lung cancer in this model. The PET/CT diagnosis model had a sensitivity and area under the curve (AUC) of 86.5% and 0.89, respectively. The testing group comprising 462 cases showed a sensitivity and AUC of 85.7% and 0.87, respectively, while the validation group comprising 229 cases showed a sensitivity and AUC of 88.4% and 0.91, respectively. Additionally, the proposed model improved the clinical discrimination ability for solid pulmonary nodules (SPNs) in the early stage significantly.

**Conclusion:**

The feature data collected from PET/CT scans can be analyzed automatically using machine learning techniques. The results of this study demonstrated that the proposed model can significantly improve the accuracy and positive predictive value (PPV) of SPNs at the early stage. Furthermore, this algorithm can be optimized into a robotic and less biased PET/CT automatic diagnosis system.

## Introduction

Lung cancer is one of the most prevalent and deadliest types of cancer worldwide. Early detection and diagnosis of lung cancer are crucial for improving patient outcomes. At present, imaging techniques such as positron emission tomography (PET) and computed tomography (CT) are primarily utilized for diagnosing early-stage lung cancer. While CT imaging is commonly used for lung cancer screening and monitoring through morphological nodule characteristics, it presents challenges in differentiating pulmonary nodules (PNs) (1). Artificial intelligence has been gradually applied to improve CT-based cancer diagnoses, with a convolutional neural network (CNN) prediction model achieving an area under the curve (AUC) of 0.71 in distinguishing malignant from benign PNs (2). Ground glass opacity (GGO) status is considered a significant prognostic and staging-classification factor that can enhance prognostic accuracy in patients with a lung cancer tumor less than 3 cm for early-stage non-small cell lung cancer (NSCLC) (3, 4). These studies present a practical and alternative approach to automatically diagnosing lung cancer based on CT-derived features rather than complicated image analysis.

For a more detailed diagnosis of suspicious PNs based on localization and biomarkers, PET/CT is preferred (5), with a 96% accuracy in identifying adrenal metastases from benign adrenal masses in oncologic patients (6). PET scanning with ^18^Fluorine-Fluorodeoxyglucose (FDG) is commonly used to generate metabolic image information (7). PET/CT features enable a more accurate localization of an area of FDG uptake to the underlying anatomical structure. Glucose derivative metabolism generates biochemical parameters, including total lesion glycolysis (TLG), metabolic tumor volume (MTV), and standardized uptake values (SUVs), such as SUV_max_ and SUV_mean_, which have shown predictive ability for NSCLC tumor differentiation (8). TLG has been suggested as an indicator of survival for advanced stage NSCLC (9), while MTV and TLG have been identified as valuable predictors for patients with metastatic pheochromocytomas and paragangliomas (10), and better prognostic measures than SUV_max_ and SUV_mean_ for NSCLC (11). Higher values of SUV_max_, MTV and TLG have been reported to be associated with a higher risk of recurrence or death for surgical NSCLC patients (12). Recently, a machine learn-based image reconstruction method was reported for the detection of FDG-positive pulmonary nodules with a sensitivity and specificity of 69.2% and 84.5%, respectively (13).

The accurate interpretation of PNs using PET/CT imaging modality is predominantly reliant on the individual expertise and knowledge of the interpreter, resulting in significant variation in the obtained results. Consequently, the need arises to establish an unbiased evaluation system for PN analysis. However, due to the potential limitations of morphology and the variability of separated biochemical signals, there is a need to develop an optimized model for the automatic diagnosis of early-stage lung cancer. To address this, the present study employed a PET/CT generated dataset to construct a diagnostic model and evaluate its efficacy in various categories and applications.

## Materials and methods

### PET/CT data collection

This study was approved by the Institutional Ethics Committee of Shanghai Pulmonary Hospital affiliated to Shanghai Tongji University (K21-317), and the requirements for written informed consent were waived for the retrospective study. Data was collected from July 2019 to May 2021, and inclusion criteria included the availability of histopathology results with defined benign or malignant pulmonary nodules, primarily in T1/T2 stage. PET/CT data was collected from 1068 patients for modeling and further analysis. Prior to the PET/CT examination, patients were instructed to fast for at least 6 hours and serum glucose levels were monitored to ensure levels were less than 110 mg/dl before administration of ^18^F-FDG. PET images were obtained using a hybrid PET/CT scanner (Biograph mCT 64, Siemens, Germany) approximately 1 hour after intravenous injection of 3.7MBq/kg of ^18^F-FDG. CT scan parameters included a tube voltage of 120kV, automatic tube current modulation, pitch of 0.8, collimation of 16 * 1.2 mm, rotation time of 0.5 seconds, and reconstruction thickness of 5.0 mm. PET scans were performed using a three-dimensional model from the skull base to the middle of the thigh, with a scan time of 1.2 minutes. PET images were reconstructed using the TrueX+TOF (ultraHD-PET) method, with a reconstructed layer thickness of 5.0 mm and interval of 3.0 mm, and were corrected for CT attenuation. All collected data were processed using Syngo via Siemens Medical Systems for post-processing to reconstruct PET, CT, and PET/CT fusion images.

### Construction of machine-learning-based model

**Figure 1:**
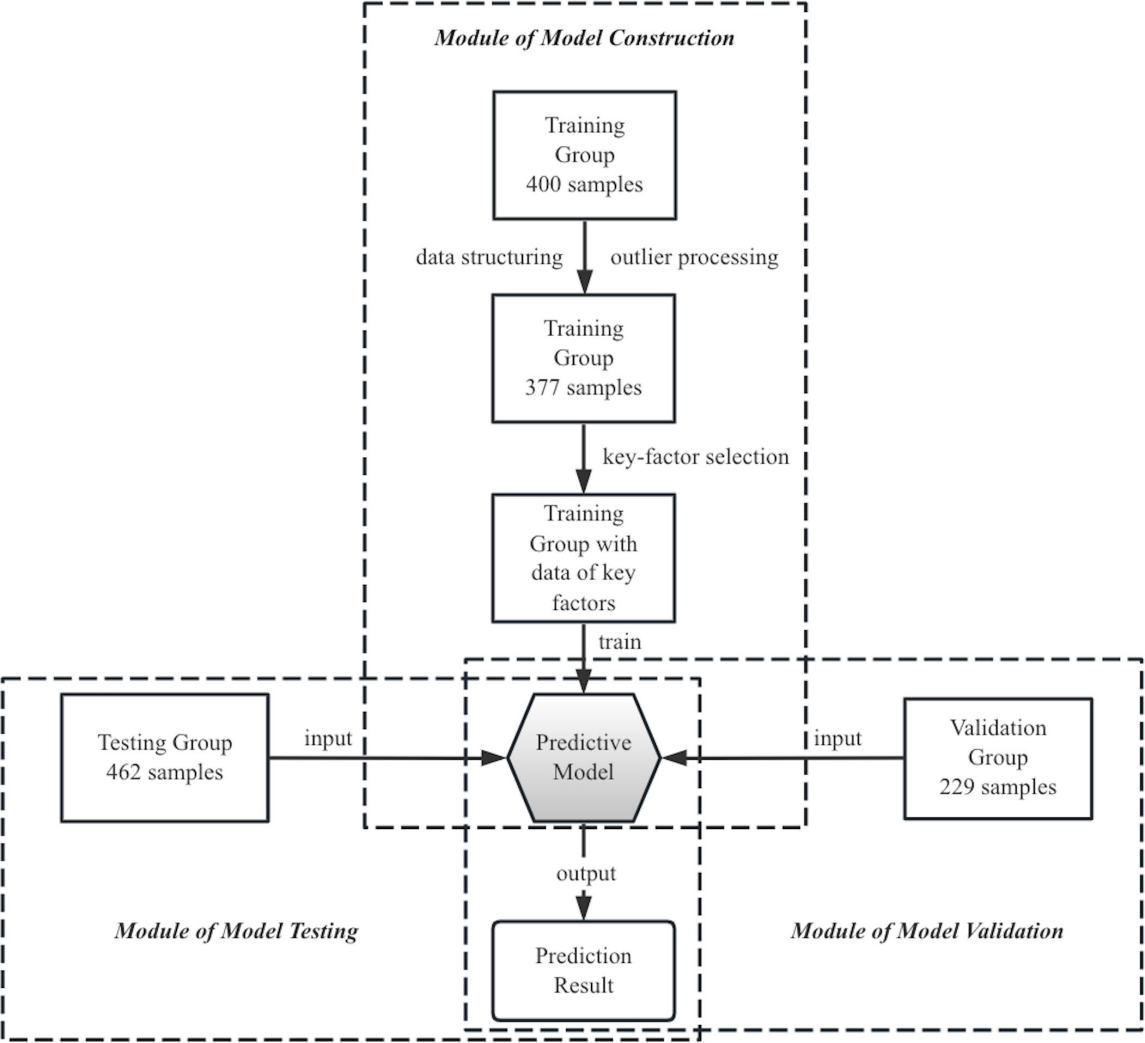
Modular description of the machine-learning-based method in this study. PET/CT images from a retrospective cohort of 200 NSCLC and 200 benign nodule patients were used as raw training data to build a predictive model, using twelve key factors. The diagnosis performance of this model was tested and validated in two separate cohorts comprising 462 and 229 patients, respectively.

The modules of machine-learning method in this study were illustrated in **Figure 1**. Prior to model training, preprocessing was implemented on raw data to ensure structural expression and exclude outliers, as previously reported (14). Afterwards, a cohort of feature data was carried out for model training. Python 3.9 software (Python Software Foundation) was used to construct and test the model. Sixteen factors comprising clinical information and PET/CT factors were selected as candidate key factors, including age, gender, smoking history, maximum diameter, lobulation, spike, calcification, hole, GGO status, upper lobe location of the PNs, SUV_max_, SUV_mean_, MTV (20%), MTV (40%), TLG (20%), and TLG (40%). Orthogonal partial least squares discrimination analysis (OPLS-DA) was used to discriminate between malignant and benign groups, and variable importance for the projection (VIP) scores were utilized to select key factors (15).

The logistic regression algorithm was applied in the study, which is suitable for modeling the probability of a certain class or event existing (16) and predicting disease risk based on several clinical characteristics (17, 18). The algorithm was trained on a dataset containing 190 benign and 187 NSCLC samples retrospectively. Mathematically, the logistic regression algorithm is represented by a standard logistic function, which is a sigmoid function that takes any real input *t* and outputs a value between zero and one (16). It takes log-odds as input and gives probability as output in terms of logit. The standard logistic function is expressed as


Eq. 1 δ(t)=1/(1+exp(−t))

where exp represents the powers of nature logarithm. It is assumed that *t* is a linear function with a set of variables

$\;x_{1},\;x_{2},\;\ldots ,\;x_{\text{n}}$ then *t* can be defined as


Eq. 2 $$t=\beta_{0}+\sum_{i=1}^{n}\beta_{i}x_{i}$$

where $\beta_{0},\;\;\beta_{1},\;\;\beta_{2},\;\ldots ,\;\beta_{\text{n}}\;$ represent the linear coefficients. The general logistic function can be written as


Eq. 3 $$p(x)=\frac{1}{1+\exp[-(\beta_{0}+\sum_{i=1}^{n}\beta_{i}x_{i})]}$$

In the logistic model, *p(x)* is indicated as the probability of positive case. By implementing the gradient descent algorithm on the training data, an optimal solution was obtained, which led to the development of the predictive model represented by Eq. 4. The result of risky score was a probability between zero and one, thus diagnosis decision could be given.


Eq. 4 risky score=1/(1+exp(−t))

Twelve key factors were finally enrolled, including gender, age, smoking history, nodule diameter, GGO status, spike, lobulation, calcification (19, 20), SUV_max_, SUV_mean_, TLG (20%), MTV (40%). For setting principles of parameters and structuring the input data, factor of age represented sample’s age in years and that of nodule diameter was the pulmonary nodules maximal diameters in millimeters. Parameters for smoking history, spike, lobulation, and calcification were assigned a value of 1 if present and 0 if absent. Gender was assigned a value of 0.6 for female samples and 0 for male samples. GGO status was assigned a value of 1 for nodule size ≥3 cm, 0 for solid nodules, and -1 for other types (e.g., ground glass, ground glass opacity, and mixed ground glass opacity). The raw data of biochemical indications including SUV_max_, SUV_mean_, TLG (20%), MTV (40%) were substituted in calculation. Coefficients of twelve key features for Eq. 4 is listed.

**Table:** 

*β*_i_	Coefficient	Feature
0	-8.422	Intercept
1	2.206	Gender
2	0.098	Age
3	-0.030	Nodule diameter
4	-0.487	Smoking history
5	1.379	Spike
6	2.265	Lobulation
7	- 4.427	Calcification
8	-2.735	GGO status
9	-0.799	SUVmax
10	1.841	SUVmean
11	-0.001	TLG(20%)
12	-0.008	MTV(40%)

### Testing and validation of the machine-learning-based model

The machine-learning-based model was tested using a dataset collected between July 2020 to December 2020, which consisted of 378 lung cancer and 84 benign samples. Equations 4-5 were used to generate diagnosis results. A validation group, comprising 147 malignant and 82 benign samples, collected between December 2020 to May 2021, was used to further evaluate the model’s performance. Fourfold tables, receiver operating characteristic curves (21) and AUC were used to evaluate the model’s performance.

### Statistical analysis

Python 3.9 and MetaboAnalyst 5.0 were used for statistical analysis and plot drawing. MetaboAnalyst 5.0, developed by members of the Wishart Research Group at the University of Alberta, is a free online tool for metabolomic data analysis. P<0.05 was considered statistically significant.

## Results

### Statistics of sample characteristics

**Figure 2:**
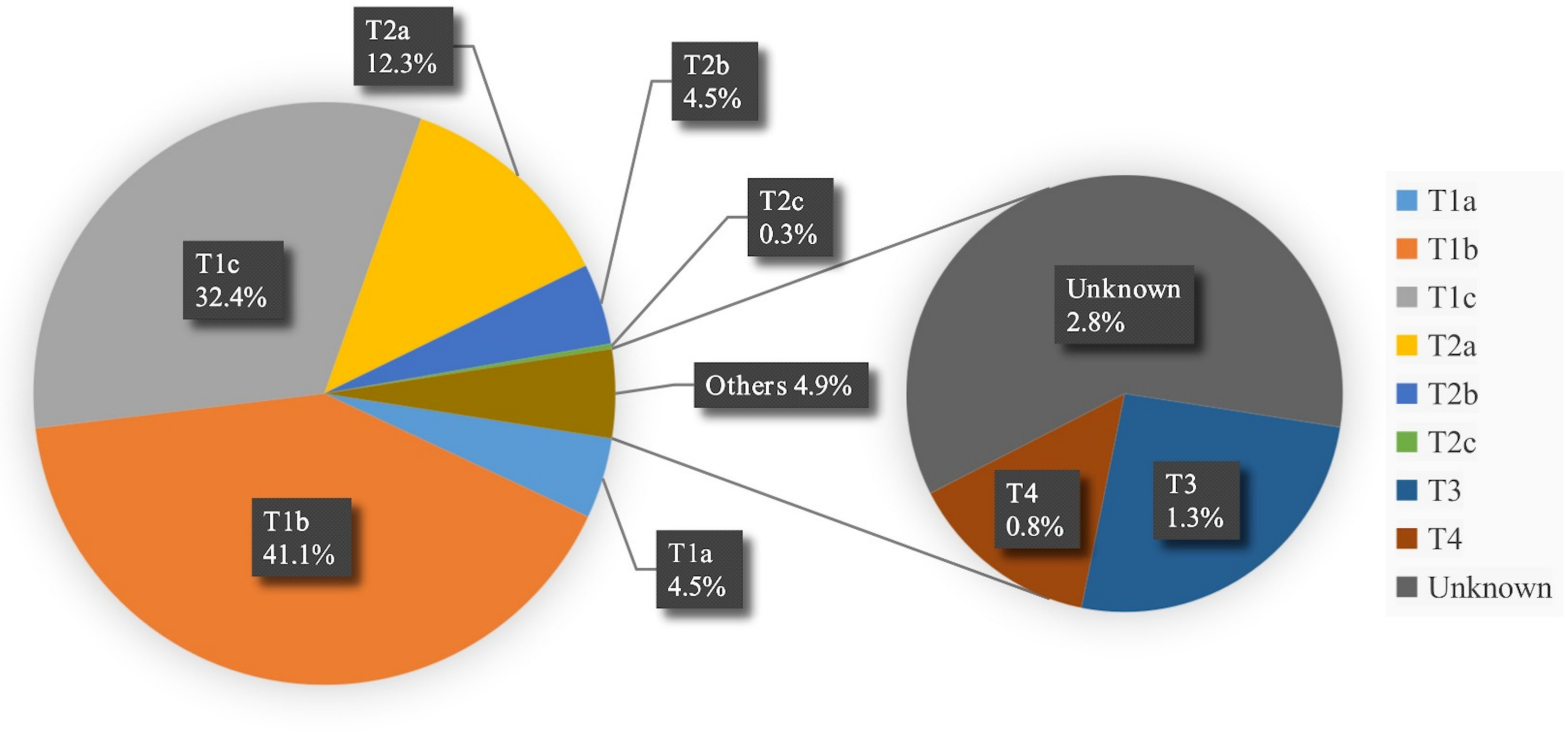
Stage statistical chart of malignant samples. * TNM system was utilized for the pathological staging of cancer in the dataset, wherein a letter or number is assigned to describe the tumor (T), node (N), and metastasis (M) categories to determine the stage.

The study enrolled a total of 1068 patients, with the stage classification of malignant samples depicted in **Figure 2**. The majority of patients were diagnosed with early-stage lung cancer, with 78% classified as T_1_N_0_M_0_ and 17.1% classified as T_2_N_0_M_0_. The patients were grouped chronologically into three sets: a retrospective training group consisting of 377 patients (35.3% of all), a testing group with 462 patients (43.3% of all), and a validation group with 229 patients (21.4% of all).

The statistical characteristics of the samples are presented in **Table 1**, which indicates no significant differences in basic clinical information between malignant and benign groups. Morphological characteristics, the average maximal diameter of PNs was also in similar ranges. However, the samples clinically diagnosed as lung cancer exhibited distinct CT features, such as GGO status, spike, calcification, lobulation, and upper lobe. In terms of biochemical characteristics, malignant PNs had higher mean values of SUV_max_, SUV_mean_, TLG (20%), and TLG (40%) but lower mean values of MTV (20%) and MTV (40%) than the benign ones.

**Table 1:** Statistical characteristics of samples recruited in the logistics regression modeling.

	Sub-type of characteristic	712 malignant samples	356 benign samples	p-value
Basic clinical characteristics	Gender (male/female)	347/365	204/152	0.004
	Age (mean/min – max)	61/26 – 84	54/15 – 81	<0.001
	Smoking history (number of samples/percentage)	255/35.8%	111/31.2%	0.066
CT morphological characteristics	Nodule diameter (mm, mean/min – max)	23.2 ± 11.4/6-128	22.3 ± 14.9/1-92	0.139
	GGO (number of GGO samples/percentage)	184/25.8%	13/3.7%	<0.001
	Spike (number of samples/percentage)	172/24.2%	23/6.5%	<0.001
	Lobulation (number of samples/percentage)	437/61.4%	76/21.3%	<0.001
	Calcification (number of samples/percentage)	5/0.7%	29/8.1%	<0.001
	Upper lobe (number of samples/percentage)	425/73.6%	171/48.0%	<0.001
	Hole (number of samples/percentage)	100/14.0%	50/14.0%	0.500
PET biochemical characteristics	SUV_max_ (mean ± std)	5.4 ± 5.1	3.4 ± 3.2	<0.001
	SUV_mean_ (mean ± std)	3.3 ± 3.1	2.0 ± 1.6	<0.001
	MTV (20%) (mean ± std)	15.1 ± 23.3	19.5± 30.7	0.005
	MTV (40%) (mean ± std)	7.4 ± 12.8	9.3 ± 15.3	0.015
	TLG (20%) (mean ± std)	46.5 ± 123.0	41.7 ± 87.1	0.257
	TLG (40%) (mean ± std)	31.7 ± 93.9	24.2 ± 52.1	0.082

### Key factors selection and modeling

**Figure 3:**
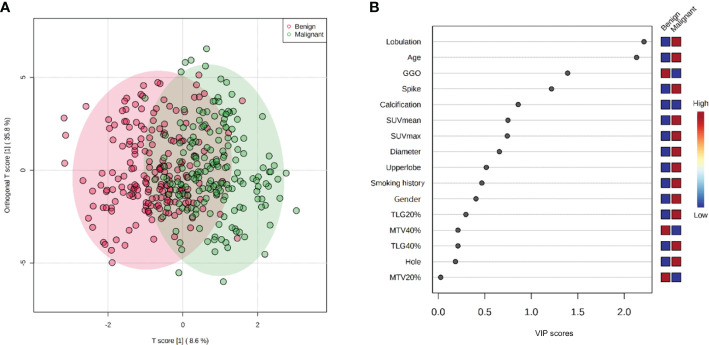
OPLS-DA and VIP score plots: **(A)** 2-D OPLS-DA score plot discriminated benign and malignant classes with inputted multivariate data. **(B)** VIP score plot showing the contribution of variables of the model. It was calculated as a weighted sum of the squared correlations between the OPLS-DA components and the original variable, which is an importance measure for variables in the OPLS-DA model. In VIP score plot, the discriminating factors are ranked in descending order of VIP score, the color boxes indicate whether factor was rising or falling (blue) in benign and malignant cases. These two plots jointly represent the effect and comparison of factors contribution for benign and malignant distinguishing.

To build a model that comprehensively reflected the influence of PET/CT parameters on lung cancer diagnosis, the parameters were evaluated synthetically. The OPLS-DA analysis showed a potential classification between the benign and non-small cell lung cancer (NSCLC) groups in the projection plot shown in **Figure 3A**. Based on the VIP score ranking in **Figure 3B**, lobulation, spike, GGO status, calcification, and the maximum diameter of PNs were considered important CT factors, while the PET indicators SUV_mean_, TLG (20%), and MTV (40%) were also included. The final predictive model, described by Eq. 4, comprised 12 key factors. The coefficients of the model indicated that spike, lobulation, SUV_mean_, and GGO status were the main contributors to lung cancer diagnosis, while calcification and SUV_max_ were more associated with benign PNs. The model achieved a sensitivity of 90.4% and specificity of 74.7% in the training cohort.

### Statistics and utilization for biochemical factors of SUV

**Figure 4:**
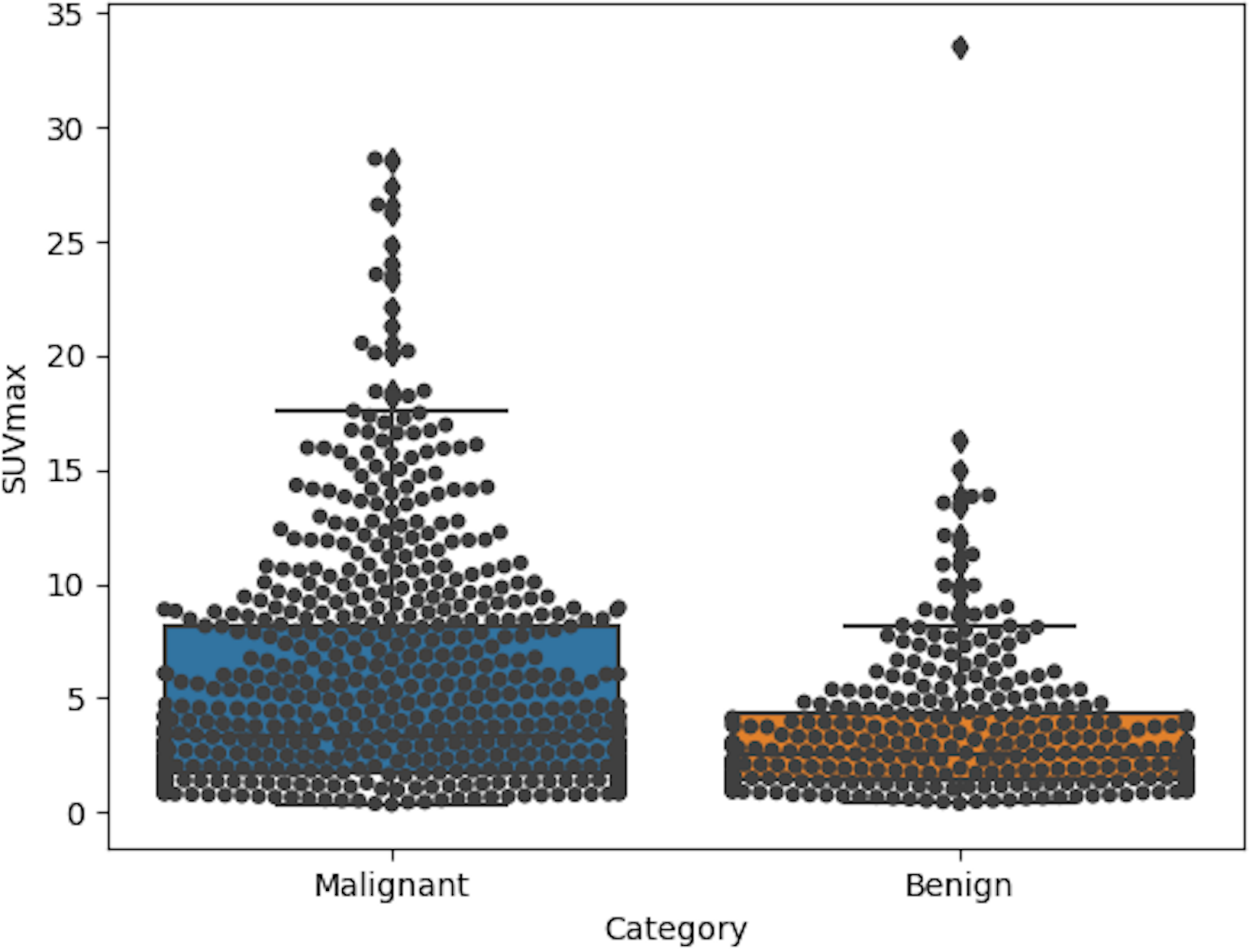
Boxplots of SUV_max_ in categories of malignant and benign for 1068 enrolled samples. In group of malignant, the mean and median value were 5.4 and 3.3, while those of benign group were about 3.4 and 2.6.

**Figure 5:**
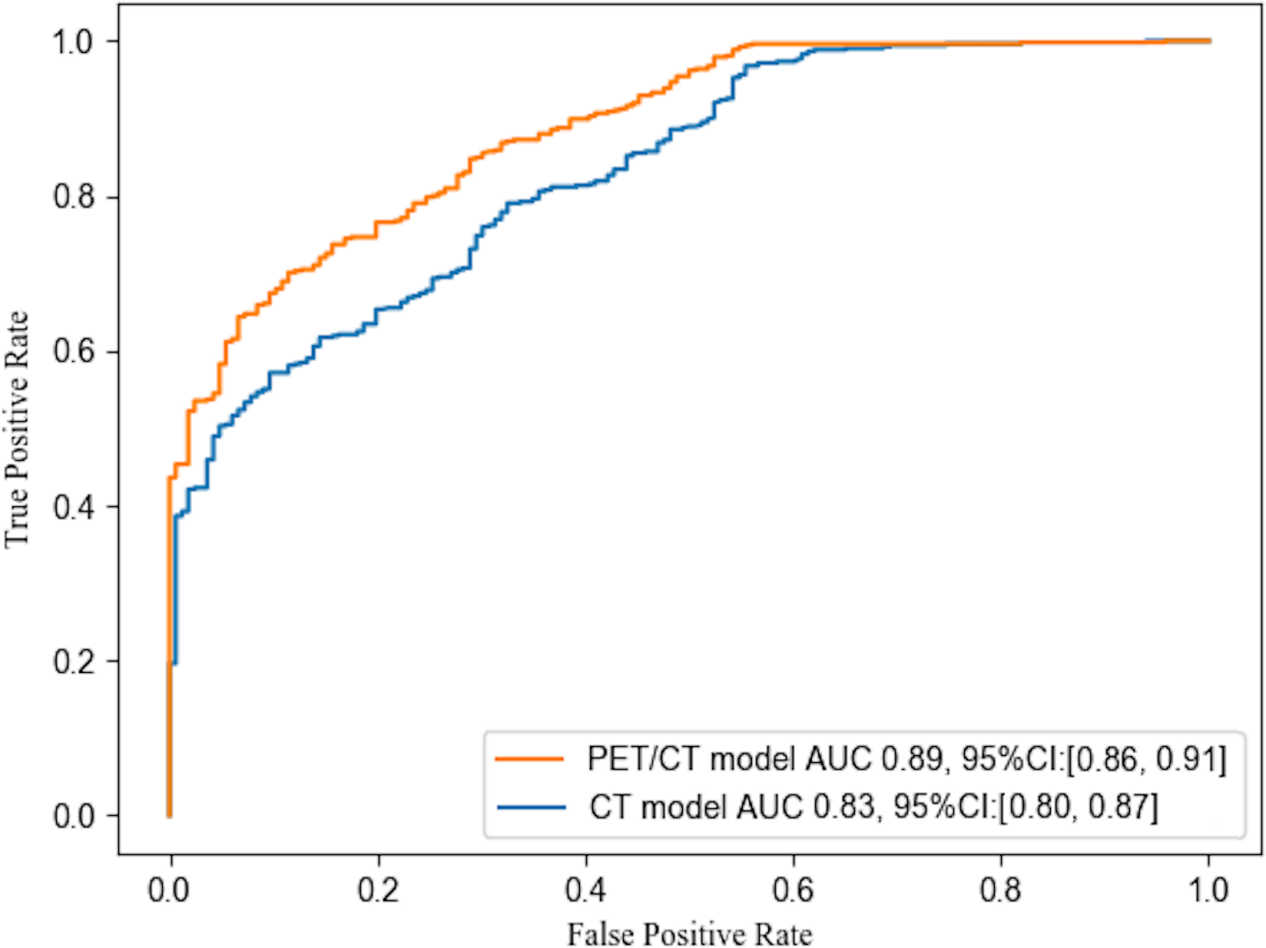
ROC curves of PET/CT model of this study and CT model without biochemical factors. The statistic included samples of testing and validation groups (with 525 lung cancer and 166 benign samples). The AUC values were 0.83 and 0.89 for the CT model and PET/CT model respectively.

Based on the model, the factor of SUV had higher VIP scores than smoking history and nodule diameter factors, and had much lower deviations than other biochemical factors (**Figure 3B**; **Table 1**), indicating its significant influence and good independence, and having expected advantages for early-stage lung cancer diagnosis. However, the commonly set threshold of SUV_max_ at around 2.5 to distinguish lung cancer from benign [if a patient had SUV_max_ higher than 2.5, it was prone to get a malignant diagnosis in clinical experience (22, 23)] was found to be insufficient, as the boxplot of benign samples showed a high probability of SUV_max_ values occurring within the range of 0 to 5 (with mean and median value of 3.4 and 2.6 shown in **Table 1** and **Figure 4**). This suggests a high risk of misdiagnosis, particularly for benign cases, if a one-size-fits-all approach is taken. Therefore, a multivariate modeling approach that takes advantage of all the biochemical factors is more appropriate for accurate diagnosis. In comparison, the PET/CT model had an AUC of 0.89, while the CT model that was trained without any biochemical factor had an AUC of 0.83, as demonstrated in **Figure 5**. These findings highlight the potential of SUV as an important biochemical factor for early-stage lung cancer diagnosis, and emphasize the importance of a multivariate modeling approach in improving the accuracy of diagnosis.

### Diagnostic performance of testing and validation groups

The present study evaluated the diagnostic performance of an automatic diagnostic model for early-stage lung cancer using a testing and validation group approach. The model’s accuracy in both groups was 82.0% and 82.1%, respectively, at the cutoff value of 0.5 (**Table 2**). Despite different ratios of malignant and benign nodule samples, the results of the model were similar to previous studies and comparable (24). The testing group provided a less biased evaluation of the model in clinical diagnosis, and together with the validation group, ensured the reliability of the results, which are important for future large-scale clinical studies.

**Table 2:** Model diagnostics performance of the training, testing and validation cohort groups.

Group	*TP	*TN	*FP	*FN	Sensitivity	Specificity	Accuracy	*PPV	*NPV	Total	Positive	Negative	*Ratio (P/N)
Training group	169	142	48	18	90.4%	74.7%	82.5%	77.9%	88.8%	377	187	190	1.0
Testing group	324	55	29	54	85.7%	65.5%	82.0%	91.8%	50.5%	462	378	84	4.5
Validation group	130	58	24	17	88.4%	70.7%	82.1%	84.4%	77.3%	229	147	82	1.8
Testing and validation groups	454	113	53	71	86.5%	68.1%	82.1%	89.5%	61.4%	691	525	166	3.2

*, TP represented number of true positive cases, TN represented number of true negative cases, FP represented number of false positive cases, FN represented number of false negative cases. PPV represented positive predictive value equaling TP/(TP+FP) *100%. NPV represented negative predictive value equaling TN/(TN+FN) *100%. Ratio of P/N was defined as number of positive/number of negative cases. Cutoff of risky score in Eq.4 was set as 0.5.

### Diagnostic performance for SPN samples

Upon statistical observation of the classifications, the testing and validation groups exhibited a pathological diagnosis of malignancy in the majority of GGO status samples. Specifically, out of 147 samples in the GGO status (GGO = -1 in the dataset), around 94.6% or 139 samples were diagnosed as malignant. Conversely, solid pulmonary nodules (SPN) samples (GGO = 0 or 1 in the dataset) had a relatively uncertain diagnostic result, with 70.8% or 386 malignancies in 545 SPN cases (**Table 3**). This trend reflects the clinical cases where patients with GGO status have a higher risk and a greater chance of being diagnosed as positive. In contrast, the SPN status introduces more ambiguity in the diagnosis (25, 26). It is notable that the conventional visual assessment of SPN on CT has a diagnostic accuracy of around 60% in distinguishing benign SPNs from malignant cases (27). Based on the data distribution and clinical difficulty, the classification of SPN warrants attention.

**Table 3:** Diagnostics performance of different sized nodules for SPN samples.

Maximal diameter of SPNs (mm)	*TP	*TN	*FP	*FN	Sensitivity	Specificity	Accuracy	*PPV	*NPV	Total	Positive	Negative	*Ratio (P/N)
≤15 mm	57	53	16	34	62.6%	76.8%	68.8%	78.1%	60.9%	160	91	69	1.3
16 – 30 mm	201	38	24	22	90.1%	61.3%	83.9%	89.3%	63.3%	285	223	62	3.6
> 30 mm	61	21	7	11	84.7%	75.0%	82.0%	89.7%	65.6%	100	72	28	2.6
All sizes of SPN samples	319	112	47	67	82.6%	70.4%	79.1%	87.2%	62.6%	545	386	159	2.4

*, same as **Table 2**.

The study involved a testing and validation group comprising 545 cases of SPN. The diagnostic model demonstrated a positive predictive value (PPV) of 87.2% and an accuracy of 79.1%, which was notably superior to the PPV of 70.8% obtained from surgical outcome (**Table 3**). The decline in PPV between the overall and SPN cohorts was evaluated, revealing that the surgical results exhibited a greater decline of 6.8% (from 76.0% to 70.8%) compared to the model, which only declined by 2.6% (from 89.5% to 87.2%), as shown in **Tables 2**, **3**.

### Comparison of different classifications of maximum diameter

Through mathematical computations, it has been observed that the varying maximum diameters of SPNs exhibit slight differences in their AUC values, with AUCs of 0.82, 0.87, and 0.89 for SPNs with maximum diameters of less than 15 mm, 16 to 30 mm, and greater than 30 mm, respectively. As the SPN diameter increases, the PPV and AUC values also increase, ranging from 78.1% to 89.7%, as presented in **Table 3**. It remains a great challenge to accurate discrimination of malignant and benign SPNs, especially in small-sized cases. In this investigation, for nodule size ≤ 15 mm, the proposed model was able to accurately diagnose 57 malignant and 53 benign nodule cases, resulting in a PPV of 78.1%. In contrast, clinical surgical findings revealed 56.9% PPV, defined 91 malignant and 69 benign cases (as shown in **Table 3**). The encouraging result of improving diagnosis PPV by 37.3% demonstrated a potential clinical applicability of this diagnosis model.

## Discussion

In this study, we utilized a machine-learning approach based on the logistic regression algorithm to develop a model for improving the diagnosis of early-stage lung cancer using PET and CT data. The featured data obtained can be automatically analyzed with minimal bias and without relying on expert knowledge. Our model performed very well in discriminating early lung nodules, especially in cases of SPN, which are considered the most challenging.

**Figure 6:**
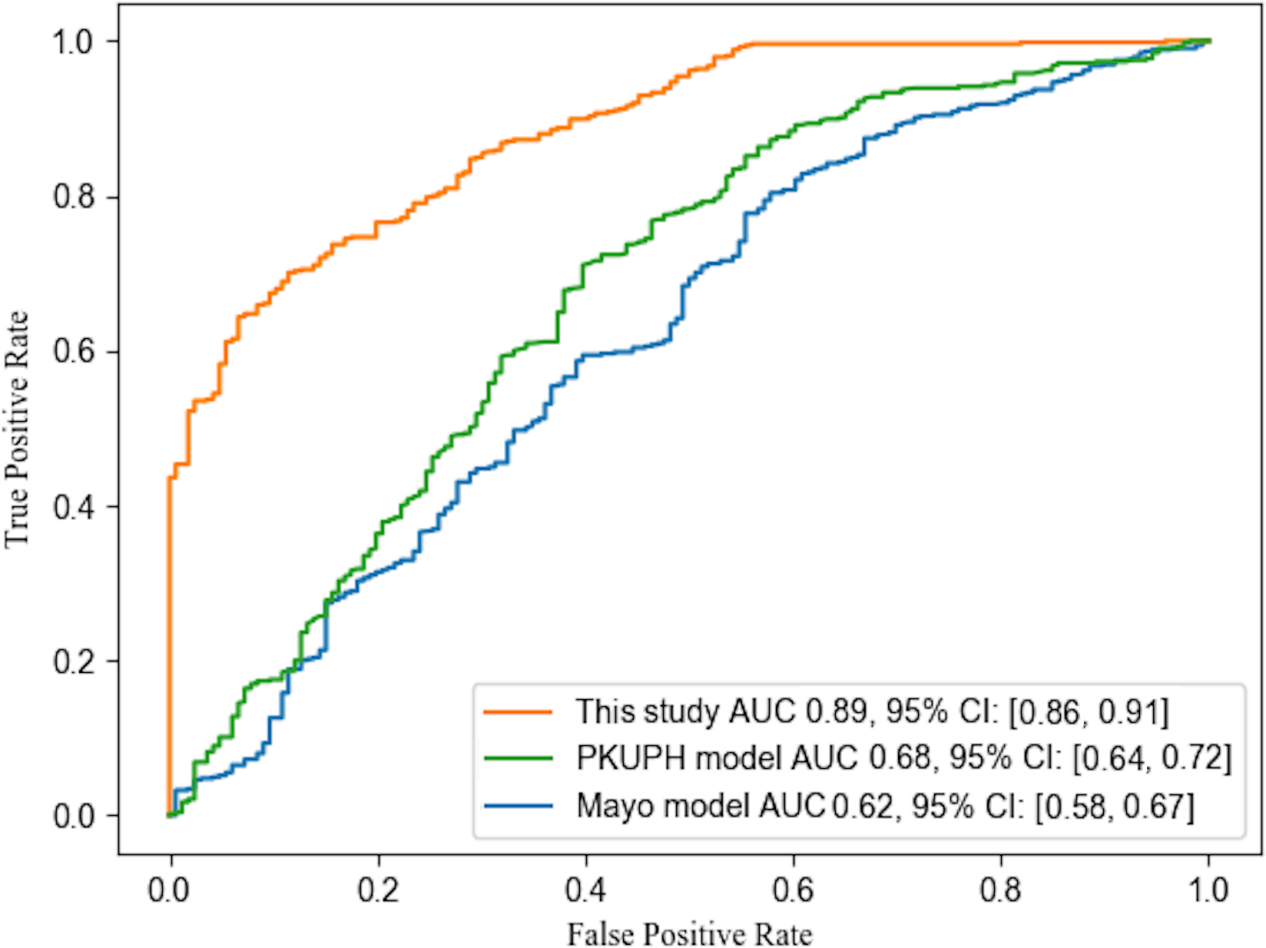
ROC curves of different models. The curves of Mayo and PKUPH model were plotted by calculating the same collection date in this study according to the published model.

We compared the overall performance of our logistic model with previous PET/CT diagnostic studies, which included 100-300 patients, and employed various methods and key factors (11, 28–32). In contrast, our study employed a much larger dataset for testing and validation consisting of 691 samples. Our results indicated that the automatic model’s diagnostic performance was comparable to those of previous studies. We present a summary of these results in **Table 4**. Several models have been proposed for the diagnosis of NSCLC based on morphological information obtained from CT and/or metabolic information from PET. Among these models, the Mayo model is a well-established method for diagnosing malignant PNs based on clinical and imaging features (1, 33). In this study, we have compared the performance of our proposed model with the Mayo model (33) and the PeKing University People’s Hospital (PKUPH) model (34). Our findings, as illustrated in **Figure 6**, demonstrate that the AUC for our proposed model was significantly higher than the AUCs for both the Mayo model (0.62) and the PKUPH model (0.68), with an AUC of 0.89. These results suggest that our model with the inclusion of biochemical factors has improved the overall performance and accuracy compared to previous models. Moreover, our model with a large sample size of early-stage lung cancer nodules has the potential to enhance the prediction of early-stage lung cancer nodules.

**Table 4:** Diagnostics performance from previous PET/CT investigations.

Study	*TP	*TN	*FP	*FN	Sensitivity	Specificity	Accuracy	PPV	NPV	Total	Positive	Negative	Ratio (P/N)	Stage	Method used
1 (29)	78	18	24	7	91.8%	42.9%	75.6%	76.5%	72.0%	127	85	42	2.0	Unknown	Predictive model
2 (31)	64	34	57	13	83.1%	37.4%	58.3%	52.9%	72.3%	168	77	81	1.0	Unknown	Predictive model
3 (28)	84	23	25	17	83.2%	47.9%	71.8%	77.1%	57.5%	149	101	48	2.1	Unknown	Visual and quantitative analysis
4 (11)	81	21	29	8	91.0%	42.0%	73.4%	73.6%	72.4%	139	89	50	1.8	Unknown	Image analysis
5 (30)	199	19	31	49	80.2%	38.0%	73.2%	86.5%	27.9%	298	248	50	5.0	Unknown	Visual and quantitative analysis
6 (32)	137	111	14	26	84.0%	88.8%	86.1%	90.7%	81.0%	288	163	125	1.3	Unknown	Quantitative analysis
Average					85.6%	49.5%	73.1%	76.2%	63.9%	–	–	–	–	–	–
This study	454	113	53	71	86.5%	68.1%	82.1%	89.5%	61.4%	691	525	166	3.2	T1/2	Predictive model

*, same as **Table 2**. Stage represented the samples’ stage of NSCLC in study.

In the testing and validation groups, which contained a total of 691 cases, experienced nuclear medicine physicians suggested 386 malignant nodules, 194 indeterminate nodules, and 111 benign nodules. Histopathology results showed that 374 and 140 nodules were truly malignant from the suggested malignant and indeterminate diagnoses, respectively, and 100 nodules were truly benign from the 111 benign diagnoses. Our model correctly identified 342 malignant and 6 benign nodules from the 386 PET/CT-diagnosed malignant nodules, 106 malignant and 32 benign nodules from the 194 PET/CT-diagnosed indeterminate nodules, and 6 malignant and 75 benign nodules from the 111 PET/CT-diagnosed benign nodules. The overall accuracy of nuclear medicine physicians was 88.9%, while that of the PET/CT model was 82.1%. This indicates the potential clinical applicability of this diagnosis model.

Clinically, various situations or departments may require different indication results, including initial diagnosis, radiology examination, preoperative examination and postoperative check, etc. This model can be easily adjusted to satisfy different clinical demands. For example, the cutoff could be adjusted as 0.7 to meet a much stricter PPV demands when the performance of positive diagnosis was concerned. A further multi-center investigation will be necessary to evaluate, optimize and finally further improve the diagnostics performance of this model.

In conclusion, a machine learning-based predictive model for diagnosis of early-stage lung cancer was created in this study with a diagnosis PPV of 89.5% and accuracy of 82.1% from testing and validation of 691 PNs. The combination of PET-derived biochemical signals with CT-derived morphological information improved the diagnosis performance of early-stage lung cancer. Additionally, the model exhibited significant discriminatory power for SPNs, thereby fulfilling certain unmet clinical demands. The automatic calculation algorithm employed by the model contributed to its robustness and reduced bias. To confirm the model, further research is required using data acquired from different PET scanners across multiple centers.

## Data availability statement

The raw data supporting the conclusions of this article will be made available by the authors, without undue reservation.

## Ethics statement

The studies involving humans were approved by the Institutional Ethics Committee of Shanghai Pulmonary Hospital affiliated to Shanghai Tongji University. The studies were conducted in accordance with the local legislation and institutional requirements. Written informed consent for participation was not required from the participants or the participants’ legal guardians/next of kin in accordance with the national legislation and institutional requirements.

## Author contributions

CH, HW, and YW contributed to conception and design of the study. YL, QLi and LZ organized the database. JH and YL performed the statistical analysis. CH wrote the first draft of the manuscript. HW, JH, QLin, JZ and HL wrote sections of the manuscript. All authors contributed to the article and approved the submitted version.
